# Prognostic analysis of *Pneumocystis jirovecii* pneumonia in patients with systemic vasculitides: a retrospective cohort study

**DOI:** 10.1007/s10067-024-07149-2

**Published:** 2024-09-21

**Authors:** Ruxuan Chen, Yujie Shi, Hongli Sun, Kai Xu, Zhiyi Li, Mengqi Wang, Chi Shao, Hui Huang

**Affiliations:** 1 Department of Pulmonary and Critical Care Medicine, Peking Union Medical College Hospital, Chinese Academy of Medical Sciences & Peking Union Medical College, #1 Shuaifuyuan Street, Dongcheng District, Beijing, 100730 China; 2 Department of Clinical Laboratory, Peking Union Medical College Hospital, Chinese Academy of Medical Sciences & Peking Union Medical College, #1 Shuaifuyuan Street, Dongcheng District, Beijing, 100730 China; 3 Department of Radiology, Peking Union Medical College Hospital, Chinese Academy of Medical Sciences & Peking Union Medical College, #1 Shuaifuyuan Street, Dongcheng District, Peking Beijing, 100730 China

**Keywords:** Antineutrophil cytoplasmic antibody–associated vasculitis, Interstitial lung disease, *Pneumocystis jirovecii* pneumonia, Systemic vasculitis

## Abstract

**Objectives:**

*Pneumocystis jirovecii* pneumonia (PJP) is a serious complication of autoimmune and inflammatory diseases. This study aimed to describe the characteristics of PJP in patients with various systemic vasculitides and explore potential prognostic factors.

**Method:**

Data on 62 enrolled PJP patients with systemic vasculitis were analyzed. Patients were stratified based on the outcomes. Prognostic factors were investigated using Cox-regression models. Characteristics of patients with and without interstitial lung disease (ILD) were compared.

**Results:**

Among 62 vasculitis-PJP patients, 48 had anti-neutrophil cytoplasmic antibody-associated vasculitis (AAV), with microscopic polyangiitis (MPA) being the most common subtype (28 patients). MPA (HR 4.33, *p* = 0.001), concomitant aspergillosis (HR 2.68, *p* = 0.019), and higher D-dimer at PJP diagnosis (HR 1.07, *p* = 0.004) were independent adverse prognostic factors for overall survival. Stable disease activity of vasculitis was an independent favorable prognostic factor (HR 0.28, *p* = 0.027). Patients with MPA were older than non-MPA patients (median age: 69 vs. 58 years, *p* = 0.001); both ILD and fibrotic ILD were more prevalent in MPA patients (ILD: 78.6% vs. 35.3%, *p* = 0.001; fibrotic ILD: 57.1% vs. 11.8%, *p* < 0.001). At the diagnosis of PJP, patients with preexisting ILD had higher counts of white cells, lymphocytes, and neutrophils, as well as higher levels of immunoglobulin (Ig) G and IgA, than patients without preexisting ILD.

**Conclusions:**

MPA was associated with a higher risk of death in patients with vasculitis-PJP, possibly due to a higher prevalence of ILD. In clinical practice, we should pay more attention to the prophylaxis and management of PJP in patients with systemic vasculitis-associated ILD and/or MPA.

**Table:** 

**Key Points** • *Data from this study showed that MPA was the most common subtype of vasculitis among vasculitis-PJP patients*. • *Compared with non-MPA patients in this study, patients with MPA were older, had more ILD and fibrotic ILD, and had a poorer prognosis*. • *In clinical practice, we should pay more attention to the prophylaxis and management of PJP in patients with systemic vasculitis-associated ILD and/or MPA*.

## Introduction

*Pneumocystis jirovecii* pneumonia (PJP) is an opportunistic infection that can be life-threatening in immunodeficient patients. Compared to PJP in human immunodeficiency virus (HIV)-infected patients, PJP in the non-HIV immunocompromised population has a shorter incubation period, a higher risk of respiratory failure and mortality, and more rapid disease progression [1, 2]. PJP is a serious complication of a variety of autoimmune and inflammatory diseases. Granulomatosis with polyangiitis (GPA) seems to be the most common autoimmune disease associated with PJP, with an estimated incidence of 6–20% [3]. It is recommended that all patients with anti-neutrophil cytoplasmic antibody (ANCA)-associated vasculitis (AAV) receiving rituximab, cyclophosphamide and/or high doses of glucocorticoids receive prophylaxis with trimethoprim/sulfamethoxazole (TMP/SMX) against PJP [4].

In patients with connective tissue diseases, risk factors for PJP include pulmonary disease, interstitial lung disease (ILD), lymphopenia, and the use of immunosuppressive agents [5]. ILD is a common complication of AAV, especially myeloperoxidase (MPO)-ANCA-positive AAV and microscopic polyangiitis (MPA) [6]. Usual interstitial pneumonia (UIP) is the most common pattern of AAV-ILD [7]. Honeycombing on chest computed tomography (CT), a typical sign of fibrotic ILD, was reported to be an independent survival risk factor for PJP [8].

Systemic vasculitides other than AAV (such as Takayasu arteritis, giant cell arteritis, and Behçet’s disease) are also commonly treated with high-dose glucocorticoids and other immunosuppressive agents, leading to an increased risk for PJP. Few studies have investigated the features of PJP in these patients. And few studies have focused on PJP in MPA, which has a relatively high prevalence of ILD. The aim of this study was to describe the characteristics of PJP in patients with various systemic vasculitides and explore their potential prognostic factors.

## Methods

### Patients

This retrospective cohort study enrolled 62 non-HIV primary systemic vasculitis patients who were complicated with PJP and admitted to Peking Union Medical College Hospital from January 2014 to November 2022. Medical records and imaging data from the electronic medical database were retrospectively reviewed. Patients with single-organ vasculitis, or secondary vasculitis which was associated with systemic disease or other probable etiology were excluded. Patients with positive HIV tests were also excluded.

This study was conducted in accordance with the Declaration of Helsinki and was approved by the institutional ethical review board (IRB) of Peking Union Medical College Hospital (approval number: K5596; approval date: 2024–03-29). Written informed consent from each patient was waived because our study was conducted using anonymized health care data, which met the IRB’s minimal risk waiver criteria.

### Definitions

The diagnosis and classification of systemic vasculitides were made in accordance with the 2012 International Chapel Hill Consensus Conference on the Nomenclature of Vasculitides [9]. The majority of enrolled patients were diagnosed with AAV, including MPA [10], GPA [11], eosinophilic granulomatosis with polyangiitis (EGPA) [12], and unclassified AAV. Unclassified AAV was defined as follows: (1) evidence of vasculitis affecting more than one organ; (2) positive for MPO-ANCA or antiproteinase 3 (PR3)-ANCA; and (3) not fulfilling the classification criteria for MPA, GPA, or EGPA.

The criteria for the diagnosis of confirmed PJP were as follows: (1) the presence of relevant respiratory manifestations, i.e., cough, dyspnea, and/or hypoxia; (2) new pulmonary shadows on chest CT; (3) identification of active *Pneumocystis jirovecii* (*P. jiroveci*) infection, i.e., (i) positive polymerase chain reaction (PCR) test for *P. jiroveci* in respiratory samples and a serum 1,3-β-D-glucan (βDG) > 100 pg/ml, and/or (ii) *P. jirovecii* cysts were observed at direct microscopic examination with Grocott’s methenamine silver (GMS) stain in respiratory samples. Respiratory samples included bronchoalveolar lavage fluid (BALF), aspirates, sputum, or lung tissue. The time of clinical diagnosis for PJP was defined as the day when PJP was suspected and empirical treatment was started.

Cytomegalovirus (CMV) viremia was defined as the blood viral load > 400 copies/ml.

Preexisting ILD was defined by the presence of hallmark manifestations [13] on chest CT before the onset of PJP. Fibrotic ILD was diagnosed with signs of traction bronchiolectasis, reticulation, architectural distortion, lung volume loss, and/or honeycombing [14].

### Data collection

Data on patient demographics, underlying diseases, prescribed medications, and laboratory and radiographic examinations were collected and comprehensively reviewed. Underlying pulmonary diseases other than ILD, including pulmonary vascular disease, airway diseases, and respiratory muscle paralysis, were also recorded. These pulmonary conditions might be complications or comorbidities of systemic vasculitis.

Immunosuppressants prescribed before the onset of PJP were recorded, except for those immunosuppressants that had been discontinued 1 month before the onset of PJP. The glucocorticoid dose was converted to equivalent prednisone (mg/d).

### Follow-up

Patients were followed up from the day that the diagnosis of PJP was confirmed. Follow-up data were obtained through outpatient records or telephone interviews. The primary endpoint of this study was all-cause mortality. Survival was defined as the interval (in days) between the diagnosis of PJP and death or the most recent follow-up (December 2023).

### Statistical analysis

Categorical variables are presented as percentages. Continuous variables are presented as the mean (± standard deviation (SD)) or median (interquartile range (IQR)) values. The Fisher’s exact test and Student’s *t*-test or Mann–Whitney *U* test were applied for categorical and continuous variables as appropriate. The Kaplan–Meier survival curves were plotted. Cox regression analysis was used to determine the variables associated with survival. The statistically significant variables selected via univariate analysis were subsequently assessed by multivariate analysis. A two-tailed *p* < 0.05 was considered statistically significant, and the hazard ratio (HR) and 95% confidence interval (CI) were indicated. All data were analyzed using SPSS 20.0 software (SPSS, Chicago, IL, USA).

## Results

### Baseline characteristics of enrolled PJP patients with systemic vasculitis

A total of 62 PJP patients with systemic vasculitis were included in the study. There were 35 males (56.5%) and 27 females (43.5%). A smoking history was reported by 29 patients (46.8%). The average age at diagnosis of PJP was 59.8 ± 14.7 (range: 21–83) years. The median time interval between the onset of PJP and the clinical diagnosis of PJP was 8 days (IQR: 4–17 days). Common comorbidities included hypertension (31 patients, 50.0%) and diabetes (24 patients, 38.7%). Common symptoms of PJP included fever (54 patients, 87.1%), dyspnea (55 patients, 88.7%), and cough (51 patients, 82.3%). At the time they developed PJP, 5 patients (8.1%) had pulmonary embolism. All patients showed bilateral lung opacities on chest CT, which were in peribronchial distribution in 24 cases, in subpleural distribution in 1 case, and diffusely distributed in 37 cases (59.7%). All patients had newly emerged ground-glass opacities, 22 patients developed consolidations, and 27 patients had newly emerged fine reticular opacities. In total, 50 patients (80.6%) developed respiratory failure, and 35 patients (56.5%) required invasive mechanical ventilation.

**Fig. 1:**
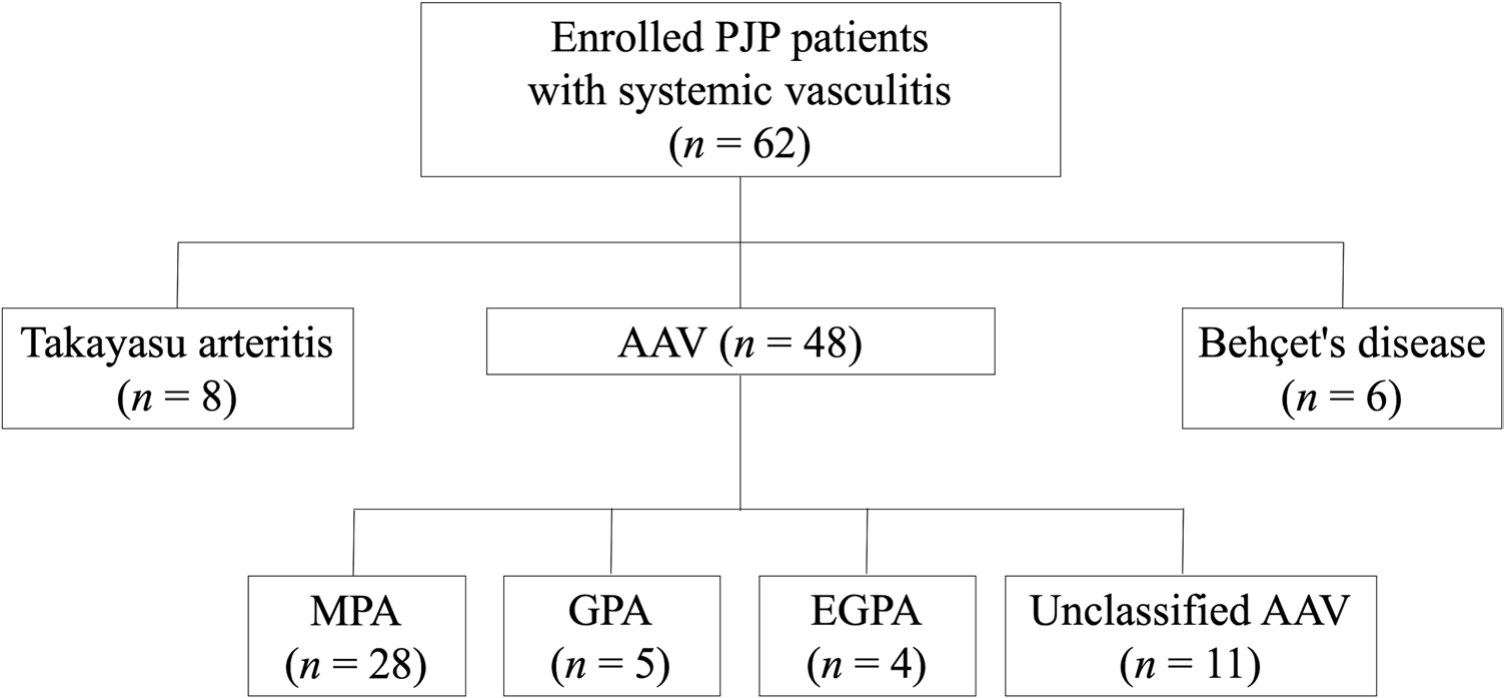
Classification of underlying vasculitis in this study. AAV, antineutrophil cytoplasmic antibody-associated vasculitis; EGPA, eosinophilic granulomatosis with polyangiitis; GPA, granulomatosis with polyangiitis; MPA, microscopic polyangiitis; PJP, *Pneumocystis jirovecii* pneumonia

The subtypes of vasculitis of the enrolled patients are shown in Fig. 1. Before PJP onset, 40 patients (64.5%) suffered from renal disease. Preexisting pulmonary diseases were present in 44 patients (71.0%), including ILD (34 patients: 22 patients with MPA, 1 patient with GPA, 3 patients with EGPA, 6 patients with unclassified AAV, and 2 patients with Behçet’s disease), bronchiectasis (3 patients), multiple pulmonary masses (2 patients, both GPA patients), pulmonary artery stenosis (2 patients), asthma (1 patient), chronic obstructive pulmonary disease (1 patient), and diaphragmatic paralysis (1 patient). Among them, there were 20 patients with fibrotic ILD, including 16 with MPA, 1 with EGPA, and 3 with unclassified AAV. In this cohort, patients with MPA were older than non-MPA patients (median age: 69 vs. 58 years, *p* = 0.001); both ILD and fibrotic ILD were more prevalent in MPA patients (ILD: 78.6% vs. 35.3%, *p* = 0.001; fibrotic ILD: 57.1% vs. 11.8%, *p* < 0.001).

All patients received glucocorticoid therapy for systemic vasculitis before the onset of PJP. The majority of patients (51 patients, 82.3%) were also treated with add-on conventional synthetic disease-modifying antirheumatic drugs (csDMARDs). The most commonly used csDMARDs in this cohort included cyclophosphamide (oral or intravenous, 31 patients) and mycophenolate mofetil (7 patients). As for biologics, rituximab was prescribed to 4 patients with AAV (3 patients with MPA and 1 patient with GPA), infliximab was given to 2 patients with Behçet’s disease, and tocilizumab was prescribed to 1 patient with Takayasu arteritis. Among the 20 patients with fibrotic ILDs, three patients were prescribed antifibrotic medication.

None of the enrolled patients had a previous history of PJP. Only 1 patient had taken primary PJP prophylaxis (TMP/SMX 160/800 mg daily) before the onset of PJP. That patient was a 74-year-old female with critical diffuse alveolar hemorrhage. She received treatment with invasive mechanical ventilation, pulse methylprednisolone, plasmapheresis, and TMP/SMX prophylaxis after being diagnosed with MPA. Her condition initially improved, but deteriorated as she was complicated with severe infections with *P. jirovecii*, CMV, and bacteria. In the end, she died of sepsis. For the other 61 patients who had not received PJP prophylaxis, possible reasons for not using PJP prophylaxis could be inferred based on medical records for 30 patients: 4 patients were allergic to TMP/SMX, 24 patients had moderate-to-severe renal dysfunction, 1 patient had liver dysfunction, and 1 patient had neutropenia.

### Characteristics of enrolled PJP patients with different clinical outcomes

The median follow-up period since the diagnosis of PJP was 66 days (IQR 28–808 days). All but four of the patients received TMP/SMX (15–20 mg/kg/day TMP) as part of the initial treatment. Among the 4 patients who were allergic to TMP/SMX, 1 patient was successfully treated with a combination of primaquine and clindamycin, while the other 3 patients were started on second-line regimens and then switched to TMP-SMX after successful desensitization.

In total, 28 patients died in this cohort. The main causes of death were respiratory failure (*n* = 18) and septic shock (*n* = 10). The all-cause mortality rates from AAV, Takayasu arteritis, and Behçet’s disease were 50% (24/48), 25% (2/8), and 33.3% (2/6), respectively. Among the AAV patients specifically, the mortality rates for MPA, GPA, EGPA, and unclassified AAV were 75% (21/28), 0, 0, and 27.3% (3/11), respectively. The detailed characteristics of patients with different clinical outcomes are shown in Table 1. Survivors were younger than nonsurvivors (54.4 vs. 66.3 years, *p* = 0.001). Preexisting ILD was more common in the nonsurvivor group (38.2% vs. 75.0%, *p* = 0.004), especially fibrotic ILD (14.7% vs. 53.6%, *p* = 0.001). Second-line antipneumocystis medications were more often used in the nonsurvivor group (35.3% vs. 67.9%, *p* = 0.011), the most common choice being caspofungin (*n* = 27). The main reason for the addition of second-line antipneumocystis medications was an inadequate response to TMP/SMX. Concomitant infections, including aspergillosis and bacterial pneumonia, were more common in the nonsurvivor group. Notably, only one of the ten patients with aspergillosis survived. The survivor group had a higher CD4/CD8 ratio (median, 1.11 vs. 0.57, *p* = 0.026). Patients in the nonsurvivor group had a higher rate of invasive mechanical ventilation requirement (100.0% vs. 20.6%, *p* < 0.001). It is worth noting that 7 of 28 nonsurvivors refused invasive mechanical ventilation although their critical condition required invasive mechanical ventilation.

**Table 1:** Characteristics of enrolled patients with different outcomes

Characteristics	Survivor group (*N* = 34)	Non-survivor group (*N* = 28)	*P* value
Age (years)	54.4 ± 15.3	66.3 ± 11.0	0.001
Male sex	17 (50.0)	18 (64.3)	0.259
Smoking history	16 (47.1)	13 (46.4)	0.961
Survival interval (days)	658 (117–1870)	28 (19–40)	< 0.001
AAV	24 (70.6)	24 (85.7)	0.156
MPA	7 (20.6)	21 (75.0)	< 0.001
Stable vasculitis	33 (97.1)	24 (85.7)	0.166
Preexisting pulmonary diseases			
ILD	13 (38.2)	21 (75.0)	0.004
Fibrotic ILD	5 (14.7)	15 (53.6)	0.001
Non-ILD pulmonary diseases^*^	7 (20.6)	3 (10.7)	0.481
Comorbidities			
Kidney diseases	20 (58.8)	20 (71.8)	0.302
Diabetes	14 (41.2)	10 (35.7)	0.660
Hypertension	17 (50.0)	14 (50.0)	1.000
Medications before PJP			
Glucocorticoid dosage^**^	41 ± 18	37 ± 16	0.366
Use of csDMARD^***^	27 (79.4)	24 (85.7)	0.518
Cyclophosphamide	14 (41.2)	17 (60.7)	0.126
Mycophenolate mofetil	4 (11.8)	3 (10.7)	1.000
Methotrexate	4 (11.8)	1 (3.6)	0.366
Rituximab	3 (8.8)	1 (3.6)	0.620
Co-infections			
CMV viremia	14 (41.2)	17 (60.7)	0.126
Aspergillosis	1 (2.9)	9 (32.1)	0.006
Bacterial pneumonia	7 (20.6)	17 (60.7)	0.001
Invasive mechanical ventilation required	7 (20.6)	28 (100.0)	< 0.001
Use of invasive mechanical ventilation	7 (20.6)	21 (75.0)	< 0.001
Mediastinal emphysema	0	6 (21.4)	0.006
PJP treatment			
Second-line regimen^#^	12 (35.3)	19 (67.9)	0.011
Clindamycin	8 (23.5)	9 (32.1)	0.449
Primaquine	2 (5.9)	3 (10.7)	0.650
Caspofungin	10 (29.4)	17 (60.7)	0.013
IVIg	13 (38.2)	13 (46.4)	0.515
Laboratory tests at the diagnosis of PJP^##^			
Lymphocytes (/μl)	500 (320–690)	400 (230–550)	0.170
Total CD19+ B cells (/μl)	24 (4–63)	9 (2–32)	0.201
Total CD4+ T cells (/μl)	162 (67–353)	61 (34–182)	0.064
Total CD8+ T cells (/μl)	148 (78–237)	103 (49–315)	0.626
CD4/CD8 ratio	1.11 (0.76–1.74)	0.57 (0.40–0.88)	0.026
Urea (mmol/l)	8.6 (5.8–16.0)	12.1 (7.9–19.6)	0.048
Albumin minimum (g/l)	27.1 ± 4.1	21.4 ± 4.7	< 0.001
Lactate dehydrogenase (U/l)	384 (300–613)	550 (438–678)	0.016
Ferritin (mg/l)	700 (352–1185)	1186 (894–2045)	0.022
D-dimer (mg/l)	1.30 (0.53–3.67)	3.61 (1.19–12.76)	0.003

Continuous variables are expressed as mean ± standard deviation or median (interquartile range). Categorical variables are *n* (%)

*AAV* anti-neutrophil cytoplasm antibody-associated vasculitis, *CMV* cytomegalovirus, *csDMARD* conventional synthetic disease-modifying antirheumatic drug, *ILD* interstitial lung disease, *IVIg* intravenous immunoglobulin, *MPA* microscopic polyangiitis, *PJP Pneumocystis jirovecii* pneumonia

^*^Non-ILD pulmonary diseases included pulmonary vascular disease, airway diseases, and respiratory muscle paralysis

^**^Glucocorticoid dose was converted to equivalent prednisone (mg/d)

^***^Immunosuppressants that were not listed in the table included calcineurin inhibitors (5 patients), azathioprine (4 patients), leflunomide (4 patients), and thalidomide (3 patients)

^#^Second-line drugs were used in combination with TMP/SMX in 30 patients and used alone in 1 patient. Second-line drugs included clindamycin, primaquine, and caspofungin in this cohort

^##^Lymphocytes subset analysis was performed in 51 patients (82.3%) at the diagnosis of PJP

**Fig. 2:**
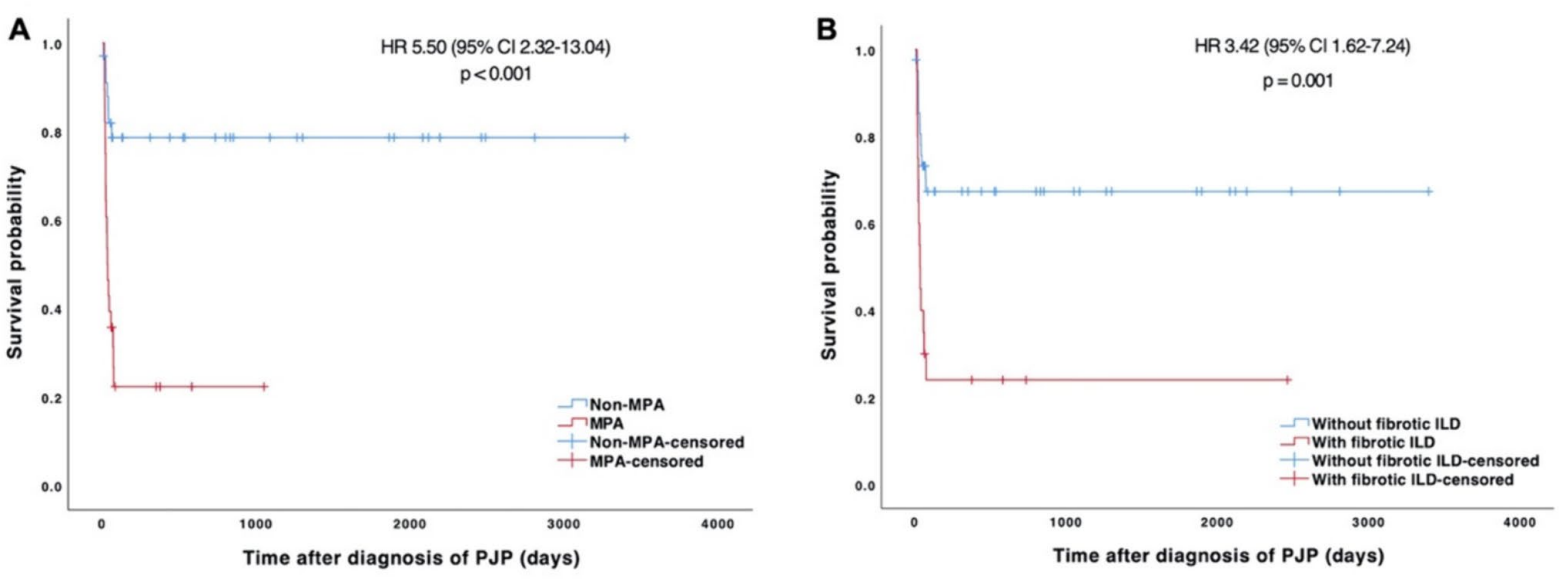
Kaplan–Meier curves for overall survival in PJP patients with systemic vasculitis in the study, stratified by MPA (**A**) and preexisting fibrotic ILD (**B**). CI, confidence interval; HR, hazard ratio; ILD, interstitial lung disease; MPA, microscopic polyangiitis; PJP, *Pneumocystis jirovecii* pneumonia

There were no significant differences between survivors and nonsurvivors in their preexisting non-ILD pulmonary diseases, immunosuppressive agents used before PJP, or CMV viremia. The survivor group had relatively higher counts of peripheral lymphocytes, CD19+ B cells, CD4+ T cells, and CD8+ T cells, though the differences did not reach statistical significance. The survivor group also had lower urea (median, 8.6 vs. 12.1 mmol/l, *p* = 0.048), higher minimum albumin (mean, 27.1 vs. 21.4 g/l, *p* < 0.001), lower lactate dehydrogenase (median, 384 vs. 550 U/l, *p* = 0.016), lower ferritin (median, 700 vs. 1186 mg/l, *p* = 0.022), and lower D-dimer (median, 1.30 vs. 3.61 mg/l, *p* = 0.003) than the nonsurvivor group. By Cox regression analysis, MPA (HR 4.33, 95% CI 1.79–10.46, *p* = 0.001), concomitant aspergillosis (HR 2.68, 95% CI 1.18–6.11, *p* = 0.019), and higher D-dimer at PJP diagnosis (HR 1.07, 95% CI 1.02–1.13, *p* = 0.004) were identified as independent adverse prognostic factors for survival (Table 2). Stable disease activity of vasculitis was an independent favorable prognostic factor (HR 0.28, 95% CI 0.09–0.87, *p* = 0.027). The Kaplan–Meier survival curves were plotted for MPA and preexisting fibrotic ILD in PJP patients with systemic vasculitis (Fig. 2).

**Table 2:** Univariable and multivariable Cox regression analyses of predictors for death in *Pneumocystis jirovecii* pneumonia patients with systemic vasculitis

Variables	Univariate analysis			Multivariable analysis		
	HR	95% CI	*P* value	HR	95% CI	*P* value
Age (years)	1.053	1.016–1.092	0.005			
MPA	5.500	2.320–13.039	< 0.001	4.325	1.788–10.463	0.001
Stable vasculitis	0.359	0.123–1.045	0.060	0.279	0.090–0.867	0.027
ILD	3.119	1.324–7.347	0.009			
Fibrotic ILD	3.422	1.618–7.236	0.001			
Aspergillosis	3.940	1.753–8.856	0.001	2.684	1.179–6.113	0.019
Bacterial pneumonia	2.831	1.314–6.098	0.008			
Use of invasive mechanical ventilation	0.680	0.318–1.453	0.319			
Mediastinal emphysema	4.199	1.674–10.536	0.002			
Use of second-line PJP regimen	2.310	1.044–5.112	0.039			
Caspofungin	2.191	1.025–4.680	0.043			
Albumin minimum (g/l)	0.877	0.818–0.941	< 0.001			
D-dimer (mg/l)	1.083	1.036–1.131	< 0.001	1.073	1.023–1.125	0.004

*CI* confidence interval, *HR* hazard ratio, *ILD* interstitial lung disease, *MPA* microscopic polyangiitis, *PJP Pneumocystis jirovecii* pneumonia

### Characteristics of enrolled PJP patients with and without preexisting ILD

Univariable Cox regression analysis found that preexisting ILD was associated with worse survival outcomes in systemic vasculitis patients who were complicated with PJP. Therefore, we further investigated differences between patients with and without preexisting ILD in this cohort (Table 3). The ILD group had an older mean age (66.6 vs. 51.5 years, *p* < 0.001), more males (76.5% vs. 32.1%, *p* < 0.001), and more patients with MPA (64.7% vs. 21.4%, *p* = 0.001), smoking history (58.8% vs. 32.1%, *p* = 0.036), diabetes (50.0% vs. 25.0%, *p* = 0.044) and concomitant infections (including CMV viremia and bacterial pneumonia, *p* = 0.011 and 0.044, respectively). Immunosuppressive agents used before PJP were comparable between the two groups, except that none of the patients in the ILD group were prescribed methotrexate. Patients in the ILD group had a higher rate of developing respiratory failure (88.2% vs. 71.4%, *p* = 0.096), invasive mechanical ventilation requirement (70.6% vs. 39.3%, *p* = 0.013), and mediastinal emphysema (17.6% vs. 0, *p* = 0.028). Concerning the immune status, patients with preexisting ILD had higher counts of white cells, lymphocytes, and neutrophils, as well as higher levels of immunoglobulin (Ig) G and IgA. The median CD4/CD8 ratio was slightly lower in the ILD group (median, 0.78 vs. 0.90, *p* = 0.554). Patients in the ILD group had lower βDG (median, 342 vs. 866 pg/ml, *p* = 0.037) and higher D-dimer (median, 4.13 vs. 1.30 mg/l, *p* = 0.003) at the diagnosis of PJP. There were no significant differences in the median value of C-reactive protein (67.5 vs. 71.0 mg/l, *p* = 0.490), erythrocyte sedimentation rate (47 vs. 55 mm/h, *p* = 0.334), ferritin (1186 vs. 1022 mg/l, *p* = 0.121), or lactate dehydrogenase (448 vs. 521 U/l, *p* = 0.432) between the two groups.

**Table 3:** Characteristics of enrolled patients with and without preexisting ILD

Characteristics	With ILD (*N* = 34)	Without ILD (*N* = 28)	*P* value
Age (years)	66.6 ± 8.4	51.5 ± 16.4	< 0.001
Male sex	26 (76.5)	9 (32.1)	< 0.001
Smoking history	20 (58.8)	9 (32.1)	0.036
Mortality	21 (61.8)	7 (25.0)	0.004
Survival interval (days)	59 (22–527)	131 (41–1291)	0.045
MPA	22 (64.7)	6 (21.4)	0.001
Comorbidities			
Kidney diseases	23 (67.6)	17 (60.7)	0.570
Diabetes	17 (50.0)	7 (25.0)	0.044
Hypertension	20 (58.8)	11 (39.3)	0.126
Medications before PJP			
Glucocorticoid dosage^*^	42 ± 17	36 ± 18	0.215
Use of csDMARD^**^	25 (73.5)	26 (92.9)	0.099
Cyclophosphamide	19 (55.9)	12 (42.9)	0.307
Mycophenolate mofetil	3 (8.8)	4 (14.3)	0.691
Methotrexate	0	5 (17.9%)	0.015
Rituximab	2 (5.9)	2 (7.1)	1.000
Co-infections			
CMV viremia	22 (64.7)	9 (32.1)	0.011
Aspergillosis	7 (20.6)	3 (10.7)	0.481
Bacterial pneumonia	17 (50.0)	7 (25.0)	0.044
Respiratory failure	30 (88.2)	20 (71.4)	0.096
Invasive mechanical ventilation required	24 (70.6)	11 (39.3)	0.013
Mediastinal emphysema	6 (17.6)	0	0.028
Second-line PJP regimen^***^	19 (55.9)	12 (42.9)	0.307
Laboratory tests at the diagnosis of PJP^#^			
White blood cell (/μl)	9020 ± 3420	6880 ± 3070	0.013
Lymphocytes (/μl)	510 (340–740)	380 (190–540)	0.023
Neutrophils (/μl)	7410 (5970–9980)	5790 (4330–6920)	0.004
Total CD19+ B cells (/μl)	17 (5–61)	16 (2–45)	0.476
Total CD3+ T cells (/μl)	303 (163–550)	225 (118–563)	0.356
CD4/CD8 ratio	0.78 (0.45–1.35)	0.90 (0.47–1.57)	0.554
IgG (g/L)	7.18 (5.57–8.37)	5.34 (4.38–7.08)	0.023
IgA (g/L)	1.86 (1.22–2.19)	1.29 (0.80–1.70)	0.022
1,3-β-D-glucan (pg/ml)	342 (75–1092)	866 (412–1757)	0.037
Lactate dehydrogenase (U/l)	448 (327–634)	521 (362–713)	0.432
D-dimer (mg/l)	4.13 (0.88–12.40)	1.30 (0.53–2.37)	0.003

Continuous variables are expressed as mean ± standard deviation or median (interquartile range). Categorical variables are *n* (%)

*CMV* cytomegalovirus, *csDMARD* conventional synthetic disease-modifying antirheumatic drug, *ILD* interstitial lung disease, *IVIg* intravenous immunoglobulin, *MPA* microscopic polyangiitis, *PJP Pneumocystis jirovecii* pneumonia

^*^Glucocorticoid dose was converted to equivalent prednisone (mg/d)

^**^Immunosuppressants that were not listed in the table included calcineurin inhibitors (5 patients), azathioprine (4 patients), leflunomide (4 patients), and thalidomide (3 patients)

^***^Second-line drugs were used in combination with TMP/SMX in 30 patients, and used alone in 1 patient. Second-line drugs included clindamycin, primaquine, and caspofungin in this cohort

^#^Lymphocytes subset analysis was performed in 51 patients (82.3%) at the diagnosis of PJP

## Discussion

Systemic vasculitides cause inflammation in blood vessel walls and usually lead to tissue injury in multiple organs. Patients with systemic vasculitides are commonly treated with powerful immunosuppressive therapy, which can compromise their immunity. Various immunosuppressants are well-established risk factors for PJP. To the best of our knowledge, this is the first study to assess the clinical characteristics and prognostic risk factors for PJP in patients with various systemic vasculitides. Our study demonstrates that MPA, concomitant aspergillosis, and higher D-dimer at PJP diagnosis were independent adverse prognostic factors for overall survival in vasculitis-PJP patients. The increased risk of death associated with MPA was possibly due to the higher prevalence of ILD.

The types of vasculitis of the enrolled PJP patients in this cohort included AAV, Behçet’s disease, and Takayasu arteritis. Few studies have reported the characteristics of PJP in patients with Behçet’s disease or Takayasu arteritis. Infection is a major contributing factor to the death of patients with AAV [15]. Although GPA reportedly put patients at higher risk of PJP than MPA or EGPA did [3], MPA was the most common type of AAV and had the highest mortality rate in this vasculitis-PJP cohort. Possible explanations include the higher prevalence of ILD in MPA patients, as ILD is a known risk factor for PJP in patients with autoimmune diseases [5, 6]. Older age might have contributed to a higher susceptibility of PJP as well.

Glucocorticoids were prescribed to all enrolled patients when they were diagnosed with systemic vasculitis before the onset of PJP. Most of them (51 patients, 82.3%) were also treated with add-on csDMARD(s), and 7 patients received biologics. Glucocorticoid is a well-recognized risk factor for PJP [16]. About 90% of PJP patients without HIV infection have received glucocorticoid therapy, usually in combination with other immunosuppressants [1]. Patients receiving high-dose corticosteroid treatment (equivalent to ≥ 20 mg of prednisone daily for ≥ 1 month) who have an additional cause of immunodeficiency (e.g., additional immunosuppressive medication) are recommended to receive PJP prophylaxis [17–19]. In this cohort, all patients received glucocorticoid therapy before PJP, and the average glucocorticoid doses in the survivor and nonsurvivor groups were both high: 41 and 37 mg of prednisone daily, respectively. The top choices of immunosuppressant in systemic vasculitis included cyclophosphamide [20]. Cyclophosphamide was the most used csDMARD in this cohort, and combination regimens including cyclophosphamide were less common in the survivor group (41.2% vs. 60.7%, *p* = 0.126). TMP/SMX is the only available choice of PJP prophylaxis in most areas of China. Common reasons for not using PJP prophylaxis included history of TMP/SMX allergy and moderate-to-severe renal dysfunction. Because renal dysfunction is not uncommon in vasculitis patients, especially AAV patients, this group of patients are at a high risk of PJP.

PJP is a serious opportunistic infectious disease. The crude mortality from treated cases of PJP in non-HIV patients is about 40% (range 8–58%), while the mortality in untreated patients is estimated to be 100% [21]. In this cohort, 35 patients (56.5%) required invasive mechanical ventilation, and 28 patients (45.2%) died. Comparisons between survivors and nonsurvivors revealed that nonsurvivors were older; had a lower CD4/CD8 ratio; had lower albumin; and had higher urea, LDH, ferritin, and D-dimer levels. The median CD4 T cell count was lower in the nonsurvivor group (61/μl vs. 162/μl, *p* = 0.064). A lower CD4 T cell count and a lower CD4/CD8 ratio reflect a worse immune status. Patients with CD4 T cell counts lower than 200/µl have a high risk for PJP in HIV-positive patients. For non-HIV patients, the cutoff value for the CD4 T cell count that warrants chemoprophylaxis has not yet been established. Li et al. [22] suggested that a CD3 T cell count < 625/μl was a predictor of PJP in the setting of autoimmune diseases, and a CD8 T cell count < 160/μl was an independent predictor of mortality. B cells also play important roles in the host immune defense against *P. jirovecii*, while B cell is an essential therapeutic target in AAV. The median CD19 B cell count was significantly decreased in this cohort (24/μl in the survivor group vs. 9/μl in the nonsurvivor group, *p* = 0.201). However, neither the total peripheral lymphocyte count nor the lymphocyte subtyping result was identified as an independent predictor of mortality in multivariable Cox regression analysis. This is probably because the prognosis is affected by various factors, including the timing of diagnosis and correction of underlying unfavorable risk factors (e.g., withholding immunosuppressants).

Apart from 4 patients who were allergic to TMP/SMX, second-line antipneumocystis medications were also used in another 27 patients, mostly in the nonsurvivor group. The main reason they were given second-line medications was an inadequate response to the initial treatment with TMP/SMX. Clinical worsening after one week of antipneumocystis treatment is a poor prognostic factor [23]. Although the preferred second-line treatment for PJP is the combination of primaquine plus clindamycin [23], primaquine was used in only 5 patients because of poor accessibility in China. Some studies have suggested that the combination of TMP/SMX with caspofungin may have synergistic antimicrobial effects against *P. jirovecii* [24, 25]. The most commonly used second-line medication in this cohort was caspofungin, but the treatment response rate (10/27, 37.0%) was not encouraging.

In case of clinical nonresponse after one week of initial anti-pneumocystis treatment, chest CT scan and bronchoalveolar lavage are suggested to identify possible secondary or co-infections [23]. PJP patients frequently have co-infections with various pathogens because of their compromised immune status. Invasive mechanical ventilation further increases their risk for secondary infections. After community-acquired and hospital-acquired bacterial pneumonia, viral infection and fungal infection are also common in this population. The common coinfected pathogens include CMV, Epstein-Barr virus, and *Aspergillus fumigatus* [26]. After multivariate Cox regression analysis, concomitant aspergillosis was identified as an independent predictor of death in this cohort. Early diagnosis and appropriate antifungal treatment might improve patient prognosis.

Preexisting pulmonary diseases (including ILD) have been recognized as risk factors for PJP in patients with connective tissue diseases [5]. In our study, preexisting ILD was associated with a higher risk of death among vasculitis-PJP patients. Further comparisons between ILD and non-ILD patients revealed that patients with preexisting ILD were older and were more likely to be male, have a smoking history, and have diabetes or concomitant infections (including CMV viremia and bacterial pneumonia). The rates of invasive mechanical ventilation and mediastinal emphysema were also higher in the ILD group. No patient in the ILD group was prescribed methotrexate, probably due to its potential pulmonary toxicity.

MPA was an independent predictor of mortality in this cohort. We found that both ILD and fibrotic ILD were more prevalent in MPA patients, consistent with previous studies [27]. Fibrotic AAV-ILD was associated with a higher risk of death than AAV with nonfibrotic ILD, and the most common cause of death in AAV-ILD patients was infection [7]. The MPA patients in this cohort were older than the non-MPA patients, so old age might have contributed to the higher mortality rate.

Notably, the immune status indicators at the diagnosis of PJP, including the peripheral lymphocyte count and serum immunoglobulin level, were higher in the ILD-PJP group. These findings suggested that patients with systemic vasculitis-associated ILD were more susceptible to PJP than were those without ILD. In clinical practice, we should pay more attention to the prophylaxis and early diagnosis of PJP in patients with systemic vasculitis-associated ILD.

This study has several limitations. First, our hospital is a tertiary referral center for rheumatic diseases and ILDs, and the ratio of complicated and/or severe cases was higher than average, which could lead to selection bias. Second, because of the retrospective nature of this study and the relatively small sample size, some of our findings were not statistically significant but implied interesting tendencies. Third, various immunosuppressants were used in our study, and the patients’ immune statuses varied, which could impact the prognosis of patients with PJP. Well-designed multicenter prospective studies are warranted to explore the prognostic factors in more depth and devise personalized treatment protocols for vasculitis-PJP.

## Conclusion

This study demonstrates that MPA, concomitant aspergillosis, and higher D-dimer at PJP diagnosis were independent adverse prognostic factors for overall survival in vasculitis-PJP patients. The increased risk of death associated with MPA was possibly due to the higher prevalence of ILD. Patients with systemic vasculitis-associated ILD were more susceptible to PJP than were those without ILD. We should pay more attention to the prophylaxis and management of PJP in patients with systemic vasculitis-associated ILD and/or MPA.

## Data Availability

The datasets generated during and/or analyzed during the current study are available from the corresponding author on reasonable request.
